# Comparison of Retzius-sparing robot-assisted laparoscopic radical prostatectomy vs standard robot-assisted radical prostatectomy: a meta-analysis

**DOI:** 10.1186/s12894-020-00685-4

**Published:** 2020-08-03

**Authors:** Yu-Li Jiang, Gao-Feng Zheng, Ze-Peng Jiang, Zhen-Li, Xie-Lai Zhou, Jin Zhou, Chun-Hua Ye, Kang-Er Wang

**Affiliations:** 1 Department of Urology, The Affiliated Hospital of Hang Zhou Normal University, Hangzhou, 310015 China; 2 School of Medicine, Hang Zhou Normal University, Hangzhou, 310016 China

**Keywords:** Prostate cancer, Retzius space, Robot-assisted radical prostatectomy, Urinary continence, Meta-analysis

## Abstract

**Background:**

To compare the postoperative continence and clinical outcomes of Retzius-sparing robot-assisted laparoscopic radical prostatectomy (RS-RALP) with non-RS RALP for patients with prostate cancer.

**Methods:**

We searched PUBMED, EMBASE and the Cochrane Central Register from 1999 to 2019 for studies comparing RS-RALP to non-RS RALP for the treatment of prostate cancer. We used RevMan 5.2 to pool the data.

**Results:**

A total of seven studies involving 1620 patients were included in our meta-analysis. No significant difference was found in positive surgical margins (PSM), bilateral nerve-sparing, postoperative hernia, complications, blood loss, or operative time. Postoperative continence was better with RS-RALP compared with non-RS RALP (OR = 1.02, OR: 2.86, 95% CI 1.94–4.20, *p* < 0.05).

**Conclusions:**

RS-RALP had a better recovery of postoperative continence than non-RS RALP. The perioperative outcomes were comparable for the two methods.

## Background

Robotic-assisted laparoscopic radical prostatectomy (RALP) has been widely used in recent times [1]. The main limitations of RALP involve the preservation of urinary continence and sexual potency and the achievement of comparable oncological outcomes (e.g., avoidance of biochemical recurrence, 5-year overall survival, and 5-year recurrence free survival) [2]. The postoperative quality of life influenced by continence, which is one of the worst complications after radical prostatectomy [3]. Several hypotheses have been proposed to explore post-RALP incontinence. The weakening of the puboperinealis from transection, traction injury, or denervation is the most important factor explaining post-RALP urinary incontinence [4]. Galfano et al. first reported that the Retzius space sparing (RS) technique during RALP was efficient in gaining good urinary continence rates while avoiding postoperative complications and yielding no positive surgical margins [5] and also reported better functional and oncological outcomes after treating 200 patients with RS-RALP [6]. RS-RALP preserves the bladder neck and urethral anatomy through a posterior plane to achieve postoperative urinary continence preservation [7]. Lim et al. reported that the RS-RALP technique was superior to the non-RS transperitoneal technique in terms of mean console time and postoperative urinary continence rates [8]. Najib et al. performed a similar meta-analysis included four studies comparing the two methods to treat with prosrare cancer [9].

However, the limitations of RS-RALP were the limited working space and its lack of feasibility in a global setting. Abu-Ghanem et al. reported that Retzius space reconstruction after transperitoneal laparoscopic robot-assisted radical prostatectomy was a better way to accelerate postoperative urinary continence, reduce early and postoperative complication rates and shorten LOS [10].

Recently, a meta-analysis conducted by Ficarra et al. indicated that posterior musculofascial reconstruction has the advantage of a one-month urinary continence recovery [11]. Recently, Sayyid et al. reported a review of the advantages of RS-RALP [12]. However, no related review or meta-analysis has addressed these issues. The aim of this systematic review was to evaluate the prevalence of and the risk factors for urinary incontinence and urinary bother, perioperative complications and short-term oncological outcomes with RS-RALP compared to non-RS RALP.

## Methods

### Search strategy

We conducted this meta-analysis according to the Preferred Reporting Items for Systematic Reviews and Meta-Analysis (PRISMA) guidelines (S1). We searched PUBMED, EMBASE and the Cochrane Central Register for studies published in English between 1999 and 2019. We used the following search terms: “Retzius-sparing” OR “Retzius preservation”, “ robot-assisted radical prostatectomy* and (‘Retzius-sparing’ OR ‘Retzius-space preservation’) AND ‘robot assisted’ AND radical AND prostatectomy”. We also used the combined Boolean operators “AND” or “OR” in the title/abstract.

### Inclusion and exclusion criteria

The inclusion criteria were as follows: (1) comparative analysis of RS-RALP with non-RS RALP for the treatment of prostate cancer; (2) studies that reported at least one of the following outcomes: postoperative continence rate, bilateral nerve-sparing rate, console time, blood loss, length of hospital stay, positive surgical margin, postoperative hernia rate, and complication rate; and (3) comparative studies of the two surgical approaches. Two investigators (YLJ and GFZ) reviewed the articles.

The exclusion criteria were as follows: (1) case reports, editorial comments, text not in English, meeting abstracts, reviews and articles without applicable data; (2) studies with insufficient data, such as those that lacked means and standard deviations; and (3) studies that were single-arm trials or were not comparative.

### Data extraction

These two authors extracted data, such as the postoperative continence rate, bilateral nerve-sparing rate, console time, blood loss, length of hospital stay, positive surgical margin, postoperative hernia rate, and complication rate. We recorded the following data: (1) baseline comparative data: study design, study size, body mass index, PSA and Gleason score; (2) intraoperative clinical outcomes: postoperative continence rate, bilateral nerve-sparing rate, console time, blood loss, length of hospital stay, positive surgical margin, postoperative hernia rate, and complication rate; and (3) postoperative complications. Any disagreements were resolved by discussion.

### Quality assessment

We used the New-Ottawa Scale (NOS) to assess the included nonrandomized studies. The NOS scores were evaluated using a 9-point system. An NOS score of 7–9 or above was considered high quality, an NOS score of 4–6 was considered medium quality, and an NOS score of 0–4 or below was considered low quality. Two reviewers (YLJ and GFZ) assessed the quality of the included studies. Table 1 presents the quality assessments of the included studies.

**Table 1:** Quality assessment of the included studies

Study	Design	Selection				Comparability	Outcome			Total
		Representativeness of exposed cohort	Selective of nonexposed Cohort	Ascertainment of exposure	Outcome not present at start		Assessment of outcome	Adequate follow-up length	Adequacy of follow-up	
Abu-Ghanem	P, S	*	*	*	*	**	*	*	*	9
Chang 2017	R, S	*	*	*	*	*	*	*	*	8
Sayyid	P, S	*	*	*	*	**	*	*	*	9
Dalela	R, S	*	*	*	*	*	*	*	*	8
Lim	P, S	*	*	*	*	**	*	*	*	9
Chang 2018	R, S	*	*	*	*	*	*	*	*	8
		Randomization	Allocation concealment		Blinding			Quality level		
Asimakopoulos	RCT	Adequate	Unclear		Unclear			Clear		
Menon	RCT	Adequate	Unclear		Unclear			Unclear		

*P* Prospectively study, *RCT* Randomised controlled trial, *R* Respectively study, *M* mutli-centers

### Statistical analysis

We used Review Manager Version 5.2 software (The Cochrane Collaboration, Oxford, UK) to perform the analysis of the included data. We used Cochran’s Q to evaluate the heterogeneity; if the value of Q < 50% or *P* > 0.01, we believed little heterogeneity was present. However, if Q > 50% and *P* < 0.01, evident heterogeneity existed. If I^2^ > 50%, the random effects model was applied. For quantitative data, we used weight mean difference (WMD) or standard mean difference (SMD) to calculate continuous data.

## Results

### Literature search

**Fig. 1:**
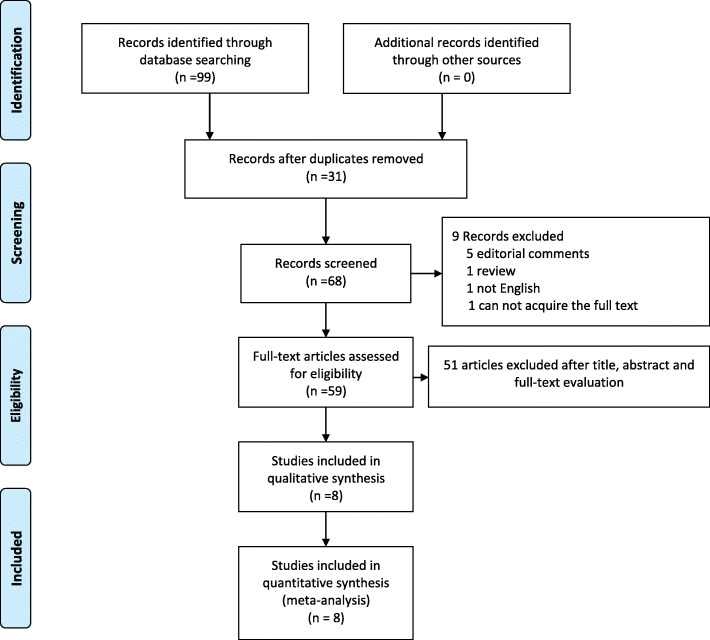
Flow diagram of the selection process of relevant studies

From the selected databases, our search obtained 99 reports. We removed 31 duplicates. After screening the titles and abstracts, 49 full texts were excluded, of which 1 report was not in English, 1 report was a review, and 5 reports were editorial comments. The remaining 59 reports underwent a comprehensive and detailed evaluation. Ultimately, 8 studies were included in this meta-analysis [8, 10, 13–18]. The process of searching studies is summarized in Fig. 1. Table 2 summarizes the baseline characteristics and assessments of the included studies.

**Table 2:** Basic Characteristics of the Included Studies

Study	Year	Design	PSA (ng/dL)		prostate volume (ml)		Hospital stay		Study group	
			RSS	Non-RS	RS	Non-RS	RS	Non-RS	RS	Non-RS
Abu-Ghanem	2017	P, S	9.7	7.2	61.1	62.7	4	44.9	51	51
Chang	2017	R, S	NA	NA	NA	NA	NA	NA	298	541
Sayyid	2017	R, S	8.75	7.07	NA	NA	NA	NA	100	100
Dalela	2017	R, S	5.7	5.4	NA	NA	NA	NA	60	60
Lim	2014	P, S	12.8	10.5	33.0	32.4	4.8	5.5	50	50
Chang	2018	R, S	18.24	12.2	40.11	41.33	NA	NA	30	30
Asimakopoulos	2018	RCT	7	6.9	NA	NA	NA	NA	39	40
Menon	2017	RCT	NA	NA	NA	NA	NA	NA	60	60

*P* Prospectively study, *RCT* Randomised controlled trial, *S* Sigle center, *R* Retrospectively study, *M* Mutli-centers, *NA* not avaliable

### Continence

**Fig. 2:**
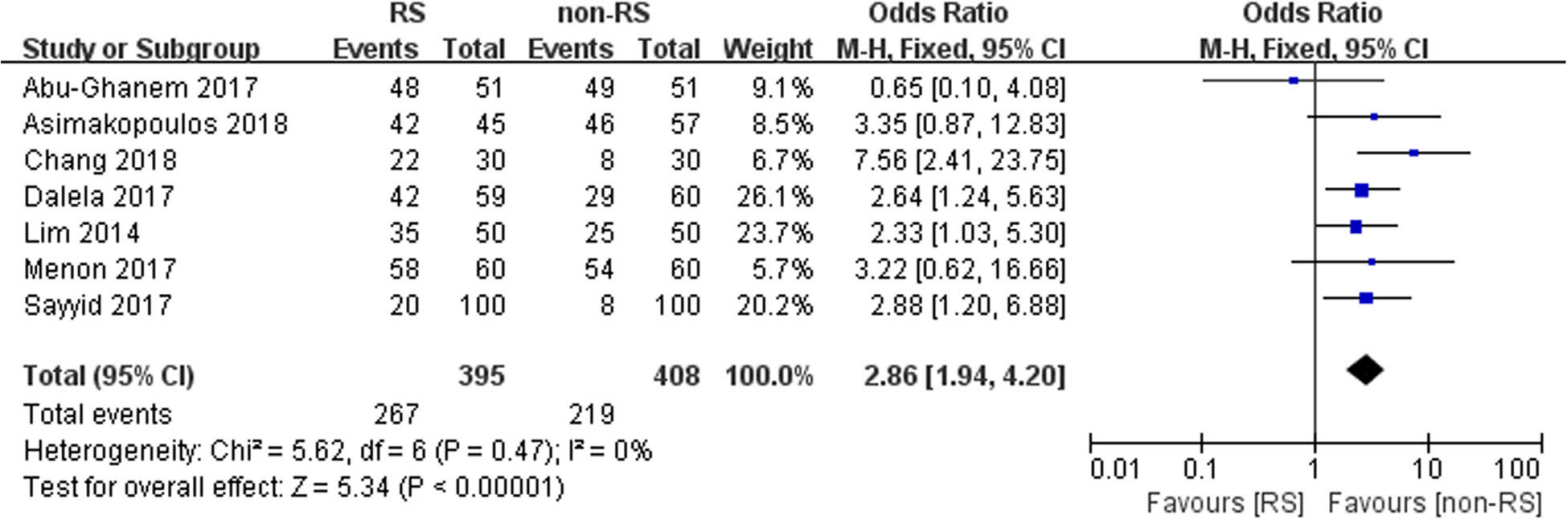
Forest plot of postoperative continence between the two groups

Seven studies reported the postoperative outcome. There was a statistically significant difference between the RS and the non-RS groups (*n* = 803, OR: 2.86, 95% CI 1.94–4.20, *p* < 0.05, I^2^ = 0, fixed-effects model, Fig. 2).

### Positive surgical margin

**Fig. 3:**
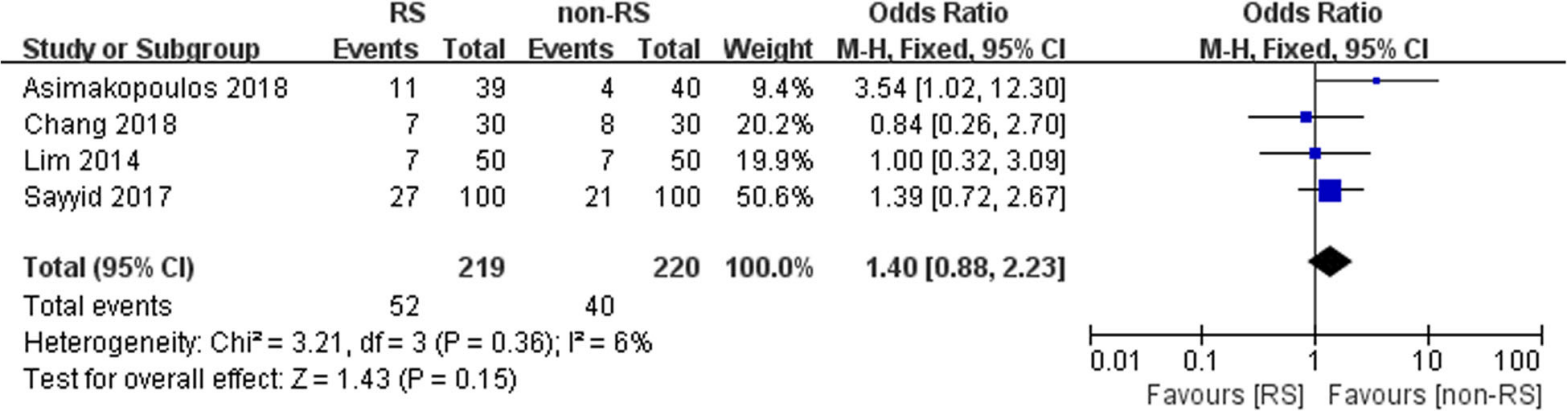
Forest plot of positive surgical margin between the two groups

Data related to positive surgical margins were obtained in four studies. No statistically significant difference between the two groups was noted (*n* = 439, OR: 1.40, 95% CI: 0.88 to 2.33, I^2^ = 6%, fixed-effects model, Fig. 3).

### Bilateral nerve-sparing

**Fig. 4:**
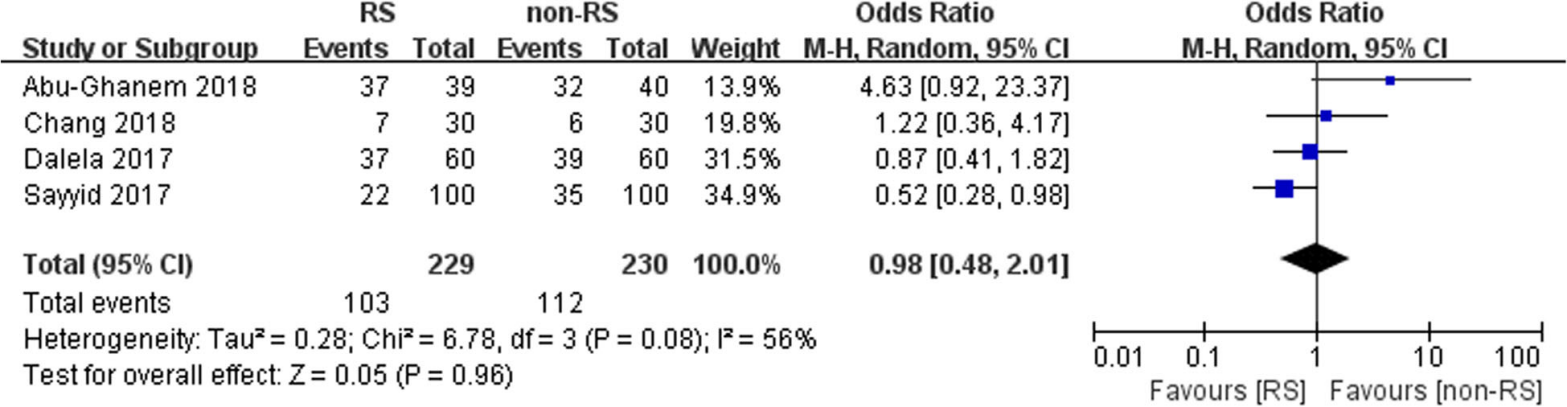
Forest plot of bilateral nerve-sparing between the two groups

Four studies included in our meta-analysis assessed bilateral nerve-sparing. Bilateral nerve-sparing was comparable between the two groups (*n* = 459 patients, OR: 0.98, 95% CI: 0.48 to 2.01, I^2^ = 56%, *p* = 0.96, random-effects model, Fig. 4).

### Postoperative hernia

**Fig. 5:**
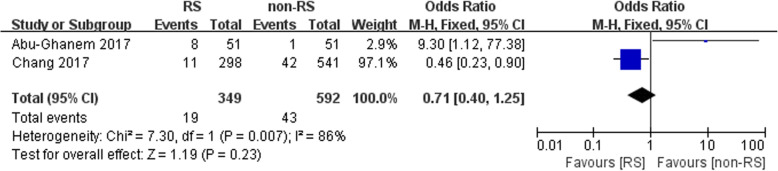
Forest plot of postoperative hernia between the two groups

Two studies were included in our meta-analysis to pool the rate of postoperative hernia. No statistically significant difference between the two groups was noted (*n* = 68, OR: 2.77, 95% CI: 0.06 to 136.11, I^2^ = 92%, random-effects model, Fig. 5).

### Complications

**Fig. 6:**
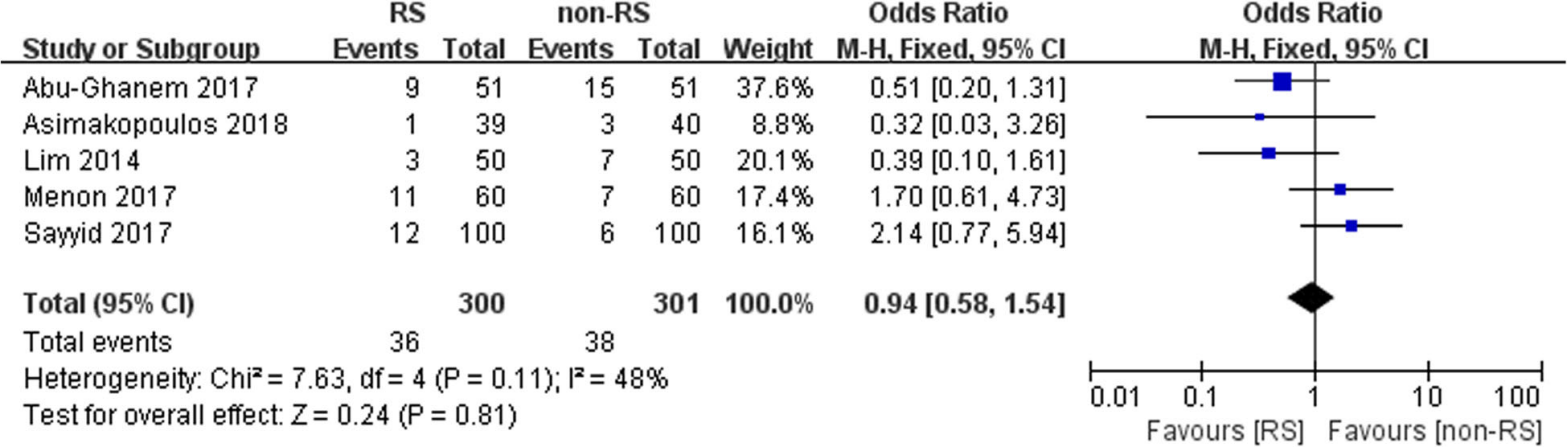
Forest plot of complications between the two groups

Four studies were included in our meta-analysis to pool the rate of postoperative complications. No statistically significant difference between the two groups was noted (*n* = 501, OR: 0.94, 95% CI: 0.58 to 1.54, I^2^ = 48%, fixed-effects model, Fig. 6).

### Blood loss

**Fig. 7:**
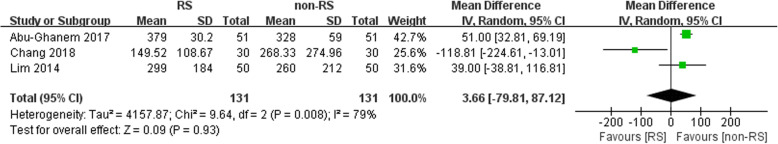
Forest plot of blood loss between the two groups

Among the three included studies reporting blood loss, no statistically significant difference between the two groups was noted (*n* = 262, WMD: 3.66, 95% CI: − 79.81 to 87.12, I^2^ = 79%, *p* = 0.93, random-effects model, Fig. 7).

### Operative time

**Fig. 8:**
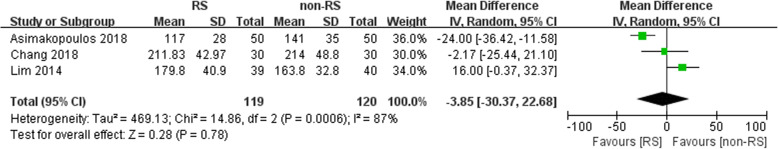
Forest plot of operative time between the two groups

Data related to the conversion rate were obtained in three studies. No statistically significant difference between the RS and non-RS groups was found (*n* = 239, WMD: -3.85, 95% CI: − 30.37 to 22.68, *p* = 0.78, random-effects model, Fig. 8).

## Discussion

In our study, we compared the postoperative continence and clinical outcomes of Retzius-sparing RALP (RS-RALP) with non-RSS RALP. No significant difference was found in the positive surgical margin (PSM), bilateral nerve-sparing, postoperative hernia, complications, blood loss, and operative time. The postoperative continence was better with RS-RALP than with non-RS RALP (*p* < 0.05).

Our study indicated that patients who underwent RS-RALP had a faster recovery of urinary continence than patients who underwent non-RS RALP (OR: 2.86, 95% CI 1.94–4.20, *p* < 0.05, Fig. 2). Similarly, Ficarra et al. performed a meta-analysis of oncological outcomes after robot-assisted radical prostatectomy and found that the one -year urinary recovery continence rate in the RALP group ranged from 84 to 97% [11]. Additionally, Chang et al. performed a study involving 60 patients (30 with RS-RALP, 30 with non-RS-RALP) and demonstrated that RS-RALP had an advantage in continence recovery over non-RS-RALP after a multivariate Cox proportional hazard regression analysis (HR: 2.461 95% CI: 1.362–4.348, *p* = 0.003) [18]. This is consistent with our results. Galfano et al. reported that the approach allows for the possibility of performing a completely intrafascial operation. During RS-RALP surgery, venosus plexus is not destroyed, thus reducing estimated blood loss [5]. In addition, the complete removal of the pubourethral ligaments is also avoided. Santok et al. conducted a retrospective study of 294 patients with low-grade prostate cancer who underwent RS-RALP. They stratified the patients into three groups according to the different tumor volumes [19]. They found that RS-RALP achieved equivalent oncological and functional outcomes for the three different prostate sizes. No significant difference was found among the three groups (< 40 ml, 40–60 ml, > 60 ml) during the 12-month follow-up (*p* = 0.25) [19]. Sayyid et al. conducted a prospective, single-center study comparing RS-RALP with conventional RALP and reported the median time to urinary continence in days (90 vs 160, *p* < 0.001) [16]. This is consistent with our meta-analysis. Ikarashi et al. reported a study that included 204 patients who underwent RALP and suggested that a preoperative membranous urethral length > 12 mm (after performing ROC analysis) was an independent predictor of postoperative urinary continence (at the 12-month follow-up) after multivariate analysis (*p* = 0.026) [20]. Porpiglia et al. reported a study involving 252 patients and indicated that the continence rate was 98.0% after catheter removal 24 wk. after RALP. The surgeons performed anterior and posterior constrictions to remodel the natural structures. In the end, the urethral-vesical anastomosis was surrounded by the anterior two layers and posterior three layers of endopelvic fascia, which restored the anatomy and covered the dorsal vascular complex (DVC) and the puboprostatic ligaments [21]. RS-RALP preserves the Retzius space and bladder neck, resulting in postoperative continence during follow-up and a faster attainment of normal urinary function compared to conventional RALP. According to Patel et al., the anterior structures provide anatomical support, allowing for a maximized urethral length for dissection and stabilizing the rhabdosphincter in its anatomical position [22]. Menon et al. performed a similar study involving 2625 patients who received RALP in which no opening of the endopelvic fascia or ligating or suturing of the DVC during the transection of the bladder neck for localized prostate cancer resulted in 95.2% of the patients being dry after catheter removal [23].

In RS-RALP, the better postoperative recovery of urinary continence was attained by avoiding the destruction of the surrounding urinary structures, thereby providing an ideal urethral length for anastomosis [24].

Lim et al. performed a study that included 50 patients who had at least 6 months of follow-up and prospectively collected patients who underwent RS-RALP and a propensity score matched conventional group that underwent conventional RALP; they found that the postoperative continence rate was 70% vs 50%, respectively (*p* = 0.039) [8]. These results are also consistent with our study. They also found that there was no significant difference between the groups of patients in both pT2 and pT3 stages (*p* = 0.54 vs *P* = 0.95, respectively) [8].

In the present study, the data regarding positive surgical margins were obtained in four studies and showed no statistically significant difference between the two groups (OR: 1.40, 95% CI: 0.88 to 2.33, Fig. 3). Similarly, Novara et al. conducted a meta-analysis indicating a similar PSM rate (RARP vs retropubic radical prostatectomy (RRP)): OR: 1.21; *p* = 0.19). They also assessed the continence of the patients in the pT2 stage and suggested that the two groups achieved a comparable PSM rate [25]. In a study by Asimakopoulos et al., 102 consecutive prostate cancer patients were prospectively randomized to TR-RALP (57) or RS-RALP (45). They also found no significant difference between the two groups.

The study by Dalela et al. involved a total of 120 consecutive patients who were assigned to receive RS-RALP. They also found that the overall PSM rate was 13% for the RS-RALP versus 25% for the non-RS RALP (*p* = 0.1) [15]. This finding is consistent with our results. However, this study had several limitations. They did not stratify patients according to the NCCN guidelines for clinically high-risk or lower-risk prostate cancer, which increased bias. The results were also influenced by the limited number of patients. Furthermore, they did not control for potential bias. Sayyid et al. reported that the PSM rate was comparable between the two groups.

Our study showed that bilateral nerve-sparing was comparable between the two groups (OR: 0.98, 95% CI: 0.48 to 2.01, *p* = 0.96, Fig. 4). Similarly, Sayyid et al. found that bilateral nerve-sparing was not associated with the surgical approach (*p* = 0.09) [16]. Additionally, Galfano et al. also concluded that the postoperative first intercourse was comparable (*p* = 0.162) [6].

In the present study, no statistically significant difference was found in postoperative hernia between the two groups (OR: 2.77, 95% CI: 0.06 to 136.11, Fig. 5). However, Abu-Ghanem et al. performed a study containing 51 patients who underwent RS-RALP and 51 patients who underwent non-RS RALP and suggested that the 12 mm port-site hernia rates were 13.7% vs 2% in the RS-RALP vs non-RS RALP (*p* = 0.03), respectively. Chang et al. conducted a retrospective study recruiting a total of 839 patients who received RALP (298 in RS-RALP vs 541 in C-RALP), demonstrating that the patients with C-RALP had a higher incidence of inguinal hernia than those with RS-RALP (79.2 vs 20.8%, respectively, *P* = 0.02) [14]. Recently, Qin et al. performed a case-control study in which 110 patients underwent RS-RALP and indicated that RS-RALP increased the recovery of urinary continence [26]. However, they found that prostate volume was an independent factor that impacted urinary continence after a multivariable regression analysis (*p* = 0.032).

Additionally, preservation of the anatomical structures of the anterior compartment, i.e., the retropubic (Retzius) space, may preserve the myopectineal orifice and its components, thereby avoiding the medial movement of the internal ring, unlike what occurs with the C-RALP technique [6, 8, 27]. They believe that the preservation of urethral support and of the anterior anatomical structures during RS-RALP resulted in a lower incidence of inguinal hernia. However, several studies reported that prostatectomy did not seem to increase the incidence of inguinal hernia after RRP. Nielson et al. and Lodding et al. reported that the retraction or stretching of the transversalis fascia or vas deferens can change the natural endopelvic fascia structure of the internal inguinal ring. This may increase the occurrence of postoperative inguinal hernia [28, 29]. Chang et al. also concluded that the postoperative incidence of inguinal hernia after radical prostatectomy is 1.8–19.4% [14]. This was consistent with our study. The different duration of postoperative follow-up could make a difference in the occurrence of inguinal hernia. However, the authors stated that 3 years were an independent factor for the occurrence of inguinal hernia.

In the present study, no statistically significant difference between the two groups was noted in terms of the complication rate (OR: 0.94, 95% CI: 0.58 to 1.54). Similarly, several studies have also reported similar results [8, 10, 17]. Postoperative urinary leakage was 11.8% vs 7.8%, (*p* = 0.5) [10]. However, Sayyid et al. also found that intraoperative complications were higher with RS-RALP (2%) vs non-RS RALP (1%) (*p* = 0.56). The RS-RALP procedure is unfamiliar to most urologists [16], which can result in the difference between the two groups.

In our present study, no statistically significant difference existed in blood loss between the two procedures (WMD: 3.66, 95% CI: − 79.81 to 87.12, *p* = 0.93, Fig. 7). Lim et al. also found that blood loss was comparable between the two groups (*p* = 0.587) [8]. Abu-Ghanem et al. also found that the patients in the RS-RALP group compared with non-RS-RALP: 328 ± 59 vs 379 ± 30.2 ml, respectively, (*p* = 0.4). This is partly due to not touching the DVC and avoiding the venous plexus [10].

Our study also found no significant difference in operative time between the RS and non-RS groups (WMD: -3.85, 95% CI: − 30.37 to 22.68, *p* = 0.78, Fig. 8). Sayyid et al. reported a similar outcome. They found that the median console time in minutes (IQR) of the RS and non-RS groups was (120.0 (105.0–142.0) vs 144.0 (118.0–171.0), respectively, *p* < 0.001) [29].

Our study had several limitations. First, the included studies were not RCTs. This can lower the confidence in our findings. Second, the surgeons worked in high-volume centers, which may not be representative of most urologists. Additionally, the included studies did not report postoperative oncological outcomes and did not have adequate follow-up. The limited included studies permitted pooling of the 5-year overall survival or recurrence-free survival. Third, the postoperative erectile function was not assessed because of the absence of mean and standard deviation values. We did not perform a cumulative analysis in our study. We did not adjust for the lack of information concerning clinical stage or biopsy parameters. The different prostate cancers and surgical procedures were independent factors determining postoperative continence and the oncological outcomes. The postoperative continence is time to event data. Due to the lack of data on the hazard ratio (HR) and standard error (SE), we could not pool the continence data into logHR and SE. This could also increase the bias. Additionally, the heterogeneity in our study could not be eliminated, and we could not perform subgroup analyses and meta-regression analyses to explore the potential heterogeneity.

## Conclusions

Our study found that RS-RALP provided a better recovery of postoperative continence than non-RS RALP. The perioperative outcomes were comparable for the two groups. More multicenter high-quality RCTs with large sample sizes are needed to verify the postoperative continence and clinical outcomes of Retzius-sparing RALP (RS-RALP) compared with non-RS RALP for patients with prostate cancer.

## Supplementary information

**Additional file 1: S1 Table.** PRISMA check list.

## Data Availability

All data generated or analyzed during this study are included in this published article.

## Abbreviations

CI: Confidence interval
WMD: Weight mean difference
OR: Odds ratio
RS: Retzius-sparing
RRP: Retropubic radical prostatectomy
RALP: Robot-assisted laparoscopic radical prostatectomy
PSM: Positive surgical margins
